# E2F-1 Directly Regulates Thrombospondin 1 Expression

**DOI:** 10.1371/journal.pone.0013442

**Published:** 2010-10-15

**Authors:** Wei Ji, Wei Zhang, Wuhan Xiao

**Affiliations:** Key Laboratory of Biodiversity and Conservation of Aquatic Organisms, Institute of Hydrobiology, Chinese Academy of Sciences, Wuhan, People's Republic of China; Institut de Génomique Fonctionnelle de Lyon, France

## Abstract

Thrombospondin 1 (TSP1) has been shown to play a critical role in inhibiting angiogenesis, resulting in inhibition of tumor growth and metastases. To figure out TSP1's regulators will lead to reveal its biological function mechanistically. In this study, we show that E2F-1 could activate the transcription of TSP1 by both promoter assays and Northern blot. Analysis of various TSP1 promoter mutant constructs showed that a sequence located −144/−137 up-stream of the transcriptional initiation site, related to the consensus E2F-responsive sequence, is necessary for the activation. In consistence with up-regulation of TSP-1 activity by over-expression of E2F-1, the knockdown of endogenous E2F-1 inhibited TSP-1 promoter activity significantly, implying that E2F-1 mediated regulation of TSP-1 is relevant *in vivo*. In addition, E2F-1 could also directly bind to the TSP1 promoter region covering −144/−137 region as revealed by ChIP assays. Furthermore, the E2F-1-induced activation of TSP1 gene transcription is suppressed by pRB1 in a dose-dependent manner. Taken together, the results demonstrate that TSP1 is a novel target for E2F1, which might imply that E2F-1 can affect angiogenesis by modulating TSP1 expression.

## Introduction

Thrombospondin 1 (TSP1) is a large oligomeric extracellular matrix glycoprotein that mediates cell-cell and cell-matrix interactions by binding cell-surface receptors including integrins, integrin associated protein (IAP)/CD47, CD36, heparin sulfate proteoglycans, low density lipoprotein related protein 1(LRP1), and very low density lipoprotein receptor (VLDLR) in addition to other extracellular matrix proteins, and cytokines [1], [2], [3], [4], [5], [6], [7], [8], [9], [10]. As the first identified naturally occurring angiogenic inhibitor, TSP1 has been shown to play a critical role in inhibiting angiogenesis, resulting in inhibition of tumor growth and metastases [11], [12], [13], [14]. In consistence with its function in anti-angiogenesis, TSP1 could inhibit endothelial cells migration *in vitro* and induce endothelial cells apoptosis *in vivo* and *in vitro* [8], [9], [10], [15], [16], [17], [18], [19]. In addition, the roles of TSP1 in wound healing, ischemia, heart remodeling, foreign body reaction, intestinal inflammation and synapse formation have been recognized [20], [21], [22], [23]. The roles of TSP1 in tumor progression are closely associated with its regulation by tumor suppressors and oncogenes. Tumor suppressors including p53, PTEN and smad4 up-regulate TSP1 expression, but the oncogenes including c-jun, v-src and c-myc down-regulate TSP1 expression [12], [24], [25], [26]. Recently, the identification of gabapentin receptor α2δ-1 as a neuronal thrombospondin receptor re-enforces TSP1's role in promoting CNS (Central nervous system) synaptogenesis [27], [28], [29]. Targeted overexpression of TSP1 in mice suppressed wound healing and tumorigenesis, while lack of functional TSP1 resulted in increased vascularization of selected tissues and significantly decreased the number of excitatory synapses [13], [21], [30]. These observations further refined TSP1's major functions *in vivo*.

Taking advantage of cell culture system and zebrafish model, we have identified that ELL could serve as a transcriptional factor to directly up-regulate TSP1 expression [31]. ELL was first identified in acute myeloid leukemia as a translocation partner of MLL. Like other MLL translocation products, the chimera MLL-ELL appears to play an important role in leukemogenesis [32], [33], [34], [35], [36]. These findings link the TSP1 with another kind of cancer progression—leukemogenesis, unrelated to its role in anti-angiogenesis.

Although the molecular mechanisms of TSP1 in anti-angiogenesis, as well as in tumor suppression, have not been well elucidated, its regulatory factors including either activators or inhibitors seem to be accounted for acting its role. Therefore, searching for more up-stream genes of TSP1 might be helpful for uncovering its roles mechanistically.

In this study, we found that E2F-1 could transactivate TSP1 promoter activity and TSP1 promoter region contains potential E2F-1 binding consensus sequences. Using promoter assays, Northern blot and ChIP (chromatin immunoprecipitation) assays, we identified that E2F-1 could directly transactivate TSP1 expression by binding to its promoter, further expending TSP1's regulatory factors.

## Materials and Methods

### Cell line and plasmid construction

HEK 293 and U2OS cells were obtained from ATCC. Both cells were maintained in Dulbecco Modified Eagle Medium (Gibco) with 10% fetal bovine serum (FBS) at 37°C in a humidified atmosphere containing 5% CO_2_.

The cDNA of human E2F1 was amplified by PCR and cloned into the pCGN-HAM (provided by William Tansey), and pCMV-tag 2C (Stratagene) by PCR. The full length cDNA of human pRB1 was amplified by RT-PCR from cDNA pool of 293 cells and subcloned into pCGN-HAM. Deletion mutants of E2F1 were generated by PCR and cloned into pCGN-HAM, pCMV-tag 2C vectors. The TSP1 promoter (−2033 to +750) luciferase reporter was described previously [31]. Deletion mutants of the TSP1 promoter were generated by restriction endonuclease digestion or PCR and subcloned into the pGL3-Basic vector (Promega). pGL3-ARF was kindly provided by Gordon Peters. pGL3-CyclinE was kindly provided by Fred Dick. pGL3-Apaf1 was cloned from total DNA of 293 cells by PCR using primers: 5′-TATCGGTACCAACAAGGCTGGGCTGTTTCCTTCC-3′ and 5′-TATCAAGCTTTACTGGACACAAAGGGAGGAGGTCTT-3′.

To accurately map the response region of E2F1 in the TSP1 promoter, four deletion mutants (−413–0, −293–0, −173–0, −113–0) were generated by PCR and subcloned into pGL3-basic vector at KpnΙ and XhoΙ sites. The primers used for PCR were: TSP1(0)-R, 5′- GATACTCGAGGGCAAGGCGGAGGAGCCGCGCGCTTTTAAAGGG-3′; TSP1(−413)-F, 5′- ATATCGGTACCTCTAGTATCCACCTCTCGCC-3′; TSP1(−293)-F, 5′- GATAGGTACCCTTGCTGATCACCCCGAGC-3′; TSP1(−173)-F, 5′- GATAGGTACCCCCGCCCCCTTCACTTTCTA-3′; and TSP1(−113)-F, 5′- GATAGGTACCGAGCCCAGACTGGCCCCCAC-3′. To further verify E2F-1 binding sites in the promoter of TSP1, on the basis of TSP1-promoter (−393–0), a series of mutation constructs (−363-mut, −238-mut, −140-mut1, −363/−238-mut, −238/−140-mut and −363/−238/−140-mut) were generated by PCR and subcloned into pGL3-basic vector at KpnΙ and XhoΙ sites. The mutant (−140-mut2) were generated on the basis of TSP1-promoter (−173-0). All the constructs were confirmed by sequencing.

To verify E2F-1's transactivity on TSP1 promoter, an artificial E2F-1 construct was generated by PCR to clone potential E2F-1 DNA binding domain (aa110–194) into pVP16 vector (Promega) to form a fusion protein with VP16 transactivation domain (VP-16-E2F-1(110–194)). The mutant of E2F-1 deficiency in DNA binding activity (E2F-1(E132)) was generated by PCR and cloned into pCMV-tag2C vector.

To confirm whether the knock-down of E2F1 has effect on TSP-1 promoter activity, the siRNA vector targeting for human E2F1 was constructed using pSUPER according to instructions [37]. The 19-nt targeting sequence for E2F1 was 5′-TATCTGTACTACGCAGCTG-3′ [38]. U2OS cells growing 6-well plate were used for verifying E2F-1 knockdown by E2F-1 siRNA using a monoclonal antibody against E2F-1(Santa Cruz). A siRNA vector targeting GFP was used as a control.

### Luciferase Reporter Assay

293 cells were grown in 24-well plates and transfected with the indicated amounts of vectors, including pTK-*Renilla* as an internal control, by Lipofectamine 2000. Luciferase activity was assayed 16–28 hr after transfection. The luciferase activity in cell extracts was determined by Dual-luciferase Reporter Assay System (Promega) according to the protocol supplied by the manufacturer. The relative light units were measured using a luminometer (Sirius, Zylux Corporation, Oak Ridge, TN). Data were normalized to *Renilla* luciferase. Data are reported as mean ± SEM of three separate experiments performed in triplicate. The statistic analysis was performed using t-test (un-paired) embedded in GraphPad Prism 5.0 Program (GraphPad Software, Inc.).

### Western Blot

Anti-GAPDH and anti-E2F1 antibodies were purchased from Santa Cruz Biotechnology and Anti-HA monoclonal antibody was purchased from Covance. Anti-Flag antibody was purchased from Sigma. Western blots were performed as described previously [31]. FujiFilm LAS4000 mini luminescent image analyzer was used to photograph blots.

### Northern Blot

293 cells were transfected with the pCMV-Myc empty vector (Clontech), or vectors expressing Myc-ELL. Total RNA was isolated with Trizol reagent (Invitrogen). Electrophoresis, transfer and hybridization were performed as described previously [31]. Briefly, the membrane was probed using synthesized oligos corresponding to human TSP1 (5′-acaagcaccacatttccagctgccat-3′) and human β-actin (5′-atgtgcaatcaaagtcctcggccaca-3′) labeled with biotin at the 3′ end. The signal was detected using the North2South Nucleus Labeling and Detection Kit (Pierce). Photography and data analysis were done as described for the Western blot analysis.

### Chromatin Immunoprecipitation (CHIP) assay

CHIP assays were performed according to the protocol described previously [31]. Briefly, 293 cells were fixed in 1% formaldehyde and then lysed in SDS buffer. Lysates were sonicated yielding DNA fragments with an average size of 200–1000 bp and precleared with protein A/G agarose. Then lysates were immunoprecipitated by 5µg of anti-E2F1 antibody (KH95) (SC-251, Santa Cruz) or normal mouse IgG (SC-2025, Santa Cruz). Antibody–nucleoprotein complex mixtures were incubated overnight and recovered by incubation with 20µl of protein A/G agarose. Washing, elution, cross-link reversal and purification of the samples for PCR analysis were performed according to the protocol described previously. The primers specific for the TSP1 promoter region (−233/0) were: 5′- AAGGCTGCGTGGGCGGGCAC-3′ (forward) and 5′-GGCAAGGCGGAGGAGCCGCGCGC-30 (reverse). The primers specific for β-actin were described previously [31].

## Results

### E2F1 activates TSP1 promoter reporter effectively

**Figure 1:**
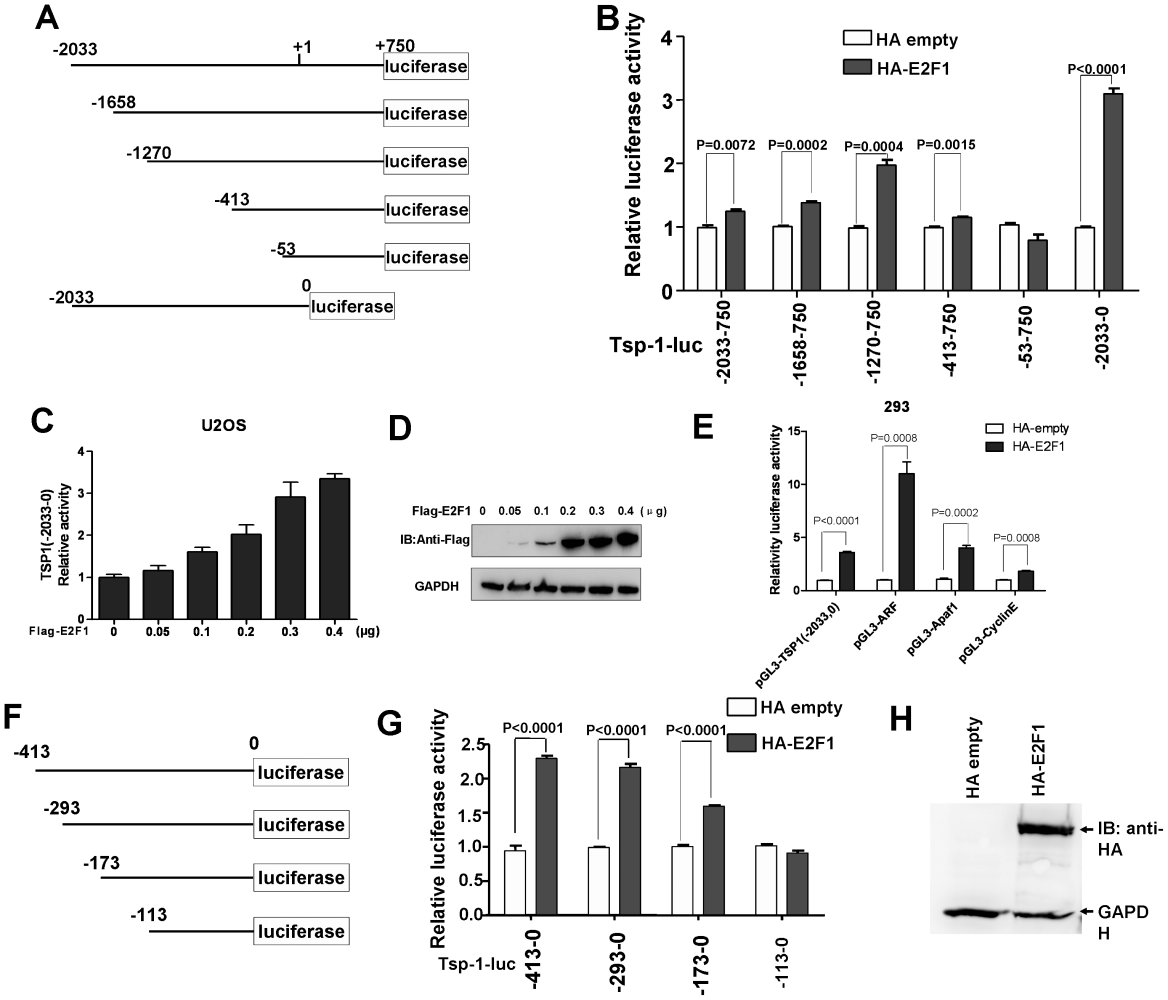
E2F-1 activates TSP1 promoter. A, schematic depiction of six TSP1 promoter deletion constructs in the region −2033 to +750bp. The transcription initial site is designated as +1. B, quantification of E2F-1 transcriptional activity on six TSP1 promoter constructs. 293 cells were used for assays and 0.2 µg HA tagged E2F-1/per well was used for transfection in 24-well plates. C, dose response of E2F1 on TSP1 promoter activity in U2OS cells. D, the expression of different doses of Flag-tagged E2F1 was verified by Western blot using anti-flag monoclonal antibody, the amounts of Flag-E2F-1 for transfection were indicated. E, the promoter luciferase reporters of E2F1 targeted gene *ARF*, *Apaf1* and *CyclinE* were used as controls to evaluate the transcriptional activity of E2F-1 on TSP1 promoter, 0.2 µg HA tagged E2F-1/per well was used for transfection in 24-well plates. F, schematic depiction of four TSP1 promoter deletion constructs in the region −413 to 0 bp. G, quantification of E2F-1 transcriptional activity on four TSP1 promoter constructs, 0.2 µg HA tagged E2F-1/per well was used for transfection in 24-well plates. H, the expression of HA-tagged E2F-1 was verified by Western blot using anti-HA monoclonal antibody.

When we performed experiments for verifying the specificity of TSP1 up-regulated by ELL using E2F-1 as a control, we found E2F-1 could also up-regulate TSP1 promoter reporter efficiently. In order to define the response elements in TSP1 promoter for E2F-1 up-regulation, initially, we did fine mapping for TSP1 promoter. Five deletion mutants of TSP1 promoter reporter were made by PCR and subcloned into pGL3-Basic vector (Fig. 1A). Subsequently, 6 different length TSP1 promoter reporter constructs were transfected into 293 cells together with the *Renilla* luciferase expression vector as an internal control in the presence of HA empty vector or HA tagged E2F-1 expression vector, a series of reporter assays were performed using Dual-luciferase Reporter Assay System (Promega) according to the protocol supplied by the manufacturer. The results showed that except for one mutant (−53–750bp), E2F-1 overexpression could activate other four TSP1 promoter significantly as revealed by statistic analysis, which suggested that E2F-1 response element was located the region between −413 to −53 (Fig. 1B). Interestingly, the TSP1 promoter reporter deleted 0–750 bp region had the highest activity stimulated by E2F-1, indicating a E2F-1 repression domain probably located in this region (0–750) (Fig. 1B). Then, we did dose response experiments for E2F-1 overexpression on TSP1 promoter activity. Flag empty vector or different dose of Flag-tagged E2F-1 expression vector, TSP1(−2033,0) promoter reporter and *Renilla* luciferase expression vector were co-transfected into U2OS cells. As shown in Fig. 1C, the TSP-1 promoter activity was steadily up-regulated along with increasing amounts of E2F1 expression vector. The expression of different amounts of Flag-tagged E2F1 was verified by Western blot (Fig. 1D). These results not only further confirmed the up-regulation of TSP-1 by E2F-1 effectively, but also ruled out the non-specific up-regulation of TSP-1 promoter activity as a result of ectopic expression of large amount of E2F-1 expression vector. Furthermore, to get a clear picture about the importance of E2F-1's effect on TSP-1 up-regulation, we chose three other well-known E2F-1 targeting genes' promoter luciferase reporters including ARF, Apaf1 and Cyclin E for evaluation. As judged by luciferase activity measurements, the transactivity of E2F-1 on TSP-1 promoter was lower than that of ARF, but higher than that of Cyclin E, which is similar to that of Apaf-1 (Fig. 1E). In addition, we did further fine domain mapping for the region −413 to 0 of TSP1 promoter (Fig. 1F). The results indicted that E2F-1 response elements were located in the region between −173 to −113 (Fig. 1G). The expression of HA-tagged E2F-1 (0.2µg vector/per well transfected into 293 cells growing in 6-well plate) was verified by Western blot using a monoclonal antibody against HA (Fig. 1H).

**Figure 2:**
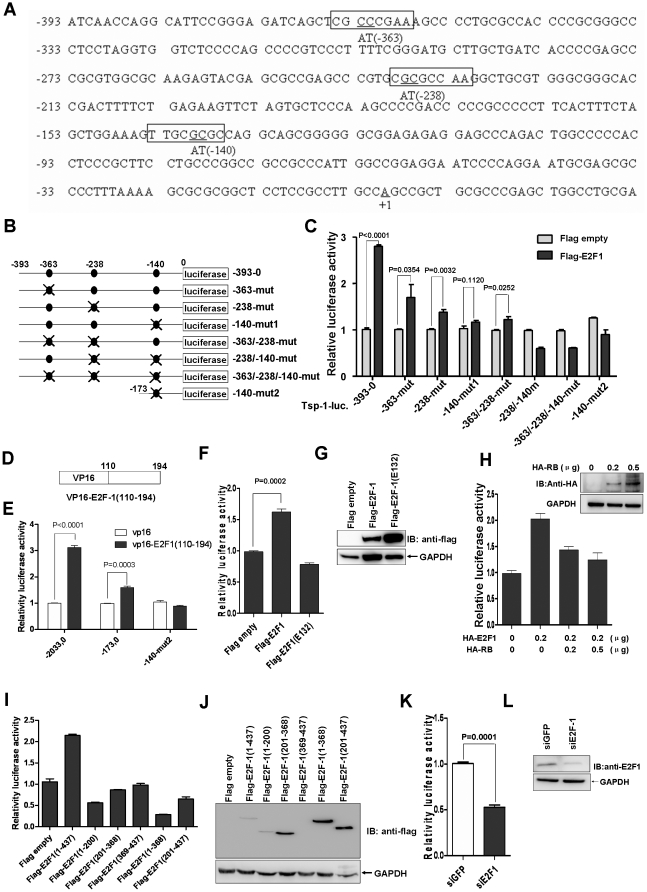
E2F-1 binding site in TSP1 promoter is required for E2F-1 up-regulation. A, the partial sequence of TSP1 promoter (−393 to +27bp). Three potential E2F-1 binding sites were marked by boxes and substitution mutations in those sites were indicated underline. B, schematic of eight TSP1 promoter mutation constructs in the region −393 to 0bp. The three potential E2F-1 binding sites were marked by dark circles and mutations were marked by “X”. C, quantification of E2F-1 transcriptional activity on eight TSP1 promoter mutation constructs, 0.2 µg Flag tagged E2F-1/per well was used for transfection in 24-well plates. D, schematic depiction of E2F-1 DNA binding region (amino acid 110–194) cloned into pVP16 vector. E, quantification of E2F-1 transcriptional activity on three TSP1 promoter mutants, 0.2 µg VP-16E2F-1(110–194) or VP-16/per well was used for transfection in 24-well plates. F, quantification of wild-type E2F-1 and E2F-1(E132) transcriptional activity on TSP1 promoter (−2033–0) luciferase reporter, 0.2 µg vector/per well was used for transfection. G, the expression of HA-tagged wild-type E2F-1 and E2F-1 DNA binding site mutant (E132) was verified by Western blot using anti-Flag monoclonal antibody. H, quantification of pRB influence on E2F-1 transcriptional activity on TSP1 promoter (−2033–0) luciferase reporter construct, the expression of HA tagged human pRB was verified by Western blot and the amounts of HA-pRB for transfection were indicated. I, quantification of E2F-1 deletion mutants on TSP1 promoter (−2033–0) luciferase reporter construct. J, the expression of flag-tagged E2F-1 deletion constructs was verified by Western blot using anti-Flag monoclonal antibody, 0.2 µg different construct/per well was used for transfection. K, quantification of E2F-1 siRNA on TSP1 promoter (−2033–0) luciferase reporter activity. L, the knockdown of E2F1 by siRNA in U2OS cells was verified by Western blot using anti-E2F1 monoclonal antibody, a siRNA targeting GFP was used as control, 0.6 µg siRNA expression vector/per well targeting E2F-1 or GFP was used for transfection in 6-well plates.

As a transcription activator, E2F1 can bind DNA cooperatively with DP proteins through the E2 recognition site (5′-TTTC[CG]CGC-3′ ) located in the promoter region of a number of genes whose products are involved in cell cycle regulation or in DNA replication [39]. After searching for the consensus sequences for E2F-1 binding, we found three potential binding sites presented in TSP1 promoter region between −363 to −137 (Fig. 2A). In order to determine whether these E2F-1 binding sites in TSP1 promoter were responsible for E2F-1 up-regulation, we made substitution mutations for the region −393 to 0 of TSP1 promoter (Fig. 2B). Then, Flag empty vector or Flag-tagged E2F1 (0.2 µg/per well in 24-well plates) was co-transfected into 293 cells along with mutant constructs and *Renilla*. The results from promoter assays showed that −363-mut and −238-mut could partially reverse the transcription activity of E2F1, but −140-mut almost completely reverse the transactivation of E2F1 (Fig. 2C). When the three putative binding sites were mutated simultaneously, the transactivation of TSP1 induced by E2F1 was abolished completely. In addition, the short mutant −140-mut2 also lost its responding for E2F-1 up-regulation, further confirming that the consensus binding site located between −144 to −137 was critical for E2F-1 responding.

To further test whether E2F1 DNA binding domain was critical for transactivating TSP1, the potential DNA binding domain (110–194 amino acids) was fused with VP16 activation domain using pVP16 vector (Clontech). Subsequently, we co-transfected VP16-E2F1 (110–194) (0.2 µg/per well in 24-well plates) along with TSP1 promoter luciferase reporters TSP1 (−2033 to 0 ), TSP1(−173 to 0) or TSP1 (−140-mut2 ) respectively. The luciferase assays showed that VP16-E2F1-(110–194) could indeed transactivate the TSP1 promoter reporters significantly (p<0.0001) but not TSP1 (−140-mut2) (Fig. 2E). Moreover, the E2F-1 mutant (E132) lacking DNA binding activity failed to transactivate TSP1 promoter (−2033 to 0) (Fig. 2F). These observations suggested that the DNA binding activity of E2F1 is required for inducing TSP1 expression. The expression of Flag-tagged wild-type E2F-1 and the mutant (E132) were verified by Western blot (Fig. 2G) using anti-Flag antibody. In addition, in order to test whether pRB1 could affect the transactivation of E2F-1 on TSP1 promoter, we transfected two different dosage of HA-tagged pRB1 (0.2µg and 0.5µg/per well in 24-well plates) together with HA-tagged E2F-1(0.2 µg/per well in 24-well plates) and performed promoter assays, pRB1 could indeed suppress the transactivity of E2F-1 on TSP1 promoter in a dose-dependent manner (Fig. 2H), which is consistent with the effect of pRB1 on other bona fide targets of E2F-1. At the same time, different function domains of E2F1 were cloned into Flag-tagged vector and co-transfected into 293 cells with TSP1 promoter luciferase reporter TSP1(−2033 to 0). The luciferase assay showed that only full length E2F1 can up-regulate TSP1 (−2033 to 0) (Fig. 2I). The expression of different function domains of E2F1 were verified by Western blot (Fig. 2J).

To figure out whether endogenous E2F1 could still have some influence on TSP1 promoter activity, we knocked down the expression of endogenous E2F-1 by an E2F1-specific short interfering RNA (siRNA) vector into U2OS cells. As expected, compared to the control (cells transfected with a siRNA vector specifically targeting GFP expression), E2F-1 knockdown could inhibit TSP-1 promoter (−2033, 0) activity significantly (p = 0.0001) (Fig. 2K). The knockdown of endogenous E2F1 was confirmed by Western blot (Fig. 2L). The observations suggested that E2F-1-mediated regulation of TSP-1 is physiologic relevant.

### E2F1 binds to the TSP1 promoter *in vivo*

**Figure 3:**
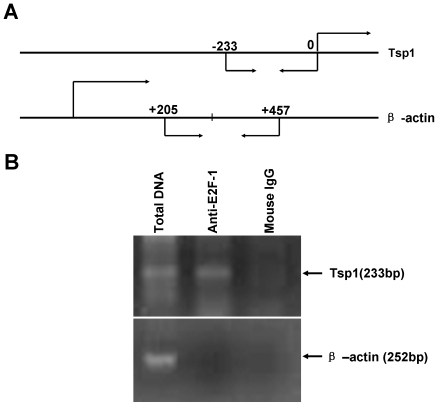
E2F-1 directly binds to TSP1 promoter. A, schematic diagram depicts the region of the TSP1 and actin genes that were amplified. The positions of PCR primers used to detect TSP1 and actin promoter fragments are indicated by arrows. B, 293 cells were treated with formaldehyde to create cross-links between E2F-1 and chromatin. The chromatin was isolated, sheared, and immunoprecipitated (IP) using monoclonal antibody against human E2F-1, or mouse IgG as control. The presence of chromatin fragments corresponding to the *TSP1* gene or to the *β-actin* gene promoter was assessed by PCR using gene-specific primers. The gel shows the recovery of TSP1 and actin gene fragments from the protein-chromatin input on the lane 1 (from left to right) as well as those recovered after immunoprecipitation with the anti-E2F-1 antibody (lane 2, up), and with mouse IgG (land 3).

To demonstrate whether the action of E2F1 on TSP1 occurs *in vivo*, we conducted chromatin Immunoprecipitation assay to test the binding of E2F1 in the TSP1 promoter. As shown in Fig. 3B, an enrichment of the TSP1 promoter was detected using anti-E2F1 antibody in 293 cells. No signal was observed using a negative control antibody (normal mouse IgG). Primers specific for β-actin was used as control. These results indicated that E2F1 could bind to the TSP1 promoter directly in 293 cells.

### E2F1 up-regulates TSP1 mRNA expression

**Figure 4:**
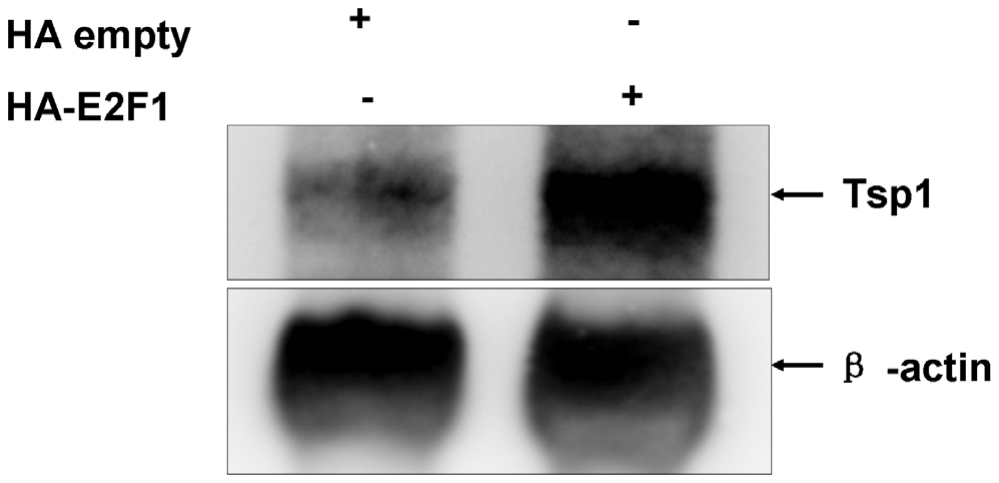
E2F-1 up-regulates TSP1 mRNA expression. Northern blot analysis of TSP1 expression in 293 cells transfected with HA empty vector of HA-E2F-1 expression vector. β-actin was used as internal control.

We next want to test whether E2F1 could up-regulate expression of the endogenous *TSP1* gene. Total RNA was extracted from 293 cells 24h after transfected by either HA-E2F1 expression vector or equivalent control empty vector. Up-regulation of TSP1 mRNA by E2F1 was confirmed by Northern blot analysis (Fig. 4) which suggested that E2F1 could indeed induce TSP1 mRNA expression.

## Discussion

In this study, through domain mapping for TSP1 promoter, we identified that the E2F-1 binding consensus sequence located in −144 to −137 of TSP1 promoter is critical for E2F1 up-regulation. Further ChIP assays confirmed that E2F-1 could bind the promoter region covering −144 to −137 *in vivo*. These observations suggested that E2F-1 could regulate TSP1 expression by directly binding to its promoter. In addition, we verified that the DNA binding domain and DNA binding ability of E2F-1 were required for transactivating TSP1 expression. Furthermore, we found that the knockdown of endogenous E2F-1 could inhibit TSP-1 promoter activity significantly, confirming the relevance of E2F-1 mediated regulation of TSP-1 *in vivo*. Taken together, these results suggested that TSP1 is a direct target of E2F-1.

E2F-1 belongs to a large family of transcription factors containing one or more conserved DNA binding domains (DBDs) that bind target promoters and regulate their expression [40], [41], [42]. To elucidate E2F-1's function, searching for E2F-1's bona fide targets has been extensively conducted. Through global gene expression profiling and promoter occupancy arrays, many target genes of E2F-1 being critical for proper cell cycle progression have been identified, which established a direct role for E2F-1 in governing cell proliferation [41], [43], [44]. Moreover, the roles of E2F-1 in cancer have been well-recognized due to its association with pRB1— a classic tumor suppressor. The pRB1 tumor suppressor directly associates with E2F-1 to modulate gene expression, either inhibit its transactivation or enhance its suppressive function [43], [44], [45], [46]. Functional inactivation of pRB1 in various human cancers leads to deregulated E2F1 activity [46]. Here, we presented data to show that pRB1 could also suppress E2F-1's transactivity on TSP1 promoter in a dose-dependent manner, which is consistent with the role of pRB1 in modulating expression of E2F-1 targets, further confirming that TSP1 serves as a bona fide target of E2F-1.

The role of genes in anti-angiogenesis represents a major aspect for their function in tumor suppression [47]. Even though, the roles of E2F-1 in tumor progression have been extensively investigated, however, to date, little is known about its function relevant to angiogenesis. TSP1 is the first identified naturally occurring angiogenic inhibitor, its role in inhibiting angiogenesis, resulting in inhibition of tumor growth and metastases has been well-defined [11], and therefore, the identification of TSP1 as a direct target of E2F-1 might open a new window for demonstrating the role of E2F-1 on anti-angiogenesis, relevant to its function in tumor suppression. Of cause, how E2F-1 acting its role in anti-angiogenesis through the regulation of TSP1 expression is definitely worth to be further investigated.
